# Comparison of different functional prediction scores using a gene-based permutation model for identifying cancer driver genes

**DOI:** 10.1186/s12920-018-0452-9

**Published:** 2019-01-31

**Authors:** Alice Djotsa Nono, Ken Chen, Xiaoming Liu

**Affiliations:** 1 Human Genetics Center, UTHealth School of Public Health, Houston, TX USA; 2 Department of Bioinformatics and Computational Biology, The University of Texas MD Anderson Cancer Center, Houston, TX USA; 3 Present Address: USF Genomics, College of Public Health, University of South Florida, Tampa, FL USA

**Keywords:** Cancer genomics, Driver genes, Function prediction method, Computational evaluation, Bioinformatics, Whole genome sequencing

## Abstract

**Background:**

Identifying cancer driver genes (CDG) is a crucial step in cancer genomic toward the advancement of precision medicine. However, driver gene discovery is a very challenging task because we are not only dealing with huge amount of data; but we are also faced with the complexity of the disease including the heterogeneity of background somatic mutation rate in each cancer patient. It is generally accepted that CDG harbor variants conferring growth advantage in the malignant cell and they are positively selected, which are critical to cancer development; whereas, non-driver genes harbor random mutations with no functional consequence on cancer. Based on this fact, function prediction based approaches for identifying CDG have been proposed to interrogate the distribution of functional predictions among mutations in cancer genomes (eLS 1–16, 2016). Assuming most of the observed mutations are passenger mutations and given the quantitative predictions for the functional impact of the mutations, genes enriched of functional or deleterious mutations are more likely to be drivers. The promises of these methods have been continually refined and can therefore be applied to increase accuracy in detecting new candidate CDGs. However, current function prediction based approaches only focus on coding mutations and lack a systematic way to pick the best mutation deleteriousness prediction algorithms for usage.

**Results:**

In this study, we propose a new function prediction based approach to discover CDGs through a gene-based permutation approach. Our method not only covers both coding and non-coding regions of the genes; but it also accounts for the heterogeneous mutational context in cohort of cancer patients. The permutation model was implemented independently using seven popular deleteriousness prediction scores covering splicing regions (SPIDEX), coding regions (MetaLR, and VEST3) and pan-genome (CADD, DANN, Fathmm-MKL coding and Fathmm-MKL noncoding). We applied this new approach to somatic single nucleotide variants (SNVs) from whole-genome sequences of 119 breast and 24 lung cancer patients and compared the seven deleteriousness prediction scores for their performance in this study.

**Conclusion:**

The new function prediction based approach not only predicted known cancer genes listed in the Cancer Gene Census (CGC), but also new candidate CDGs that are worth further investigation. The results showed the advantage of utilizing pan-genome deleteriousness prediction scores in function prediction based methods. Although VEST3 score, a deleteriousness prediction score for missense mutations, has the best performance in breast cancer, it was topped by CADD and Fathmm-MKL coding, two pan-genome deleteriousness prediction scores, in lung cancer.

**Electronic supplementary material:**

The online version of this article (10.1186/s12920-018-0452-9) contains supplementary material, which is available to authorized users.

## Background

The genetic backgrounds of cancers are highly heterogeneous [1], with almost 719 genes currently known as causally implicated in cancer etiology or development [2, 3], some genes are associated with more than one cancer type and this list is far from complete. Over the last decade, due to falling cost of high throughput sequencing, whole genome sequencing analysis has begun to take the place of exome sequencing as the method of choice for investigating genetic variants. It is widely known that in cancer genomics somatic mutations are assumed to occur randomly; however, not all these mutations are involved in carcinogenesis. Pathogenic driver mutations provide growth advantage to cancer cells; whereas, nonpathogenic passenger mutations occurring during tumorigenesis may or may not have functional effect, but play no role in cancer. Cancer driver genes (CDGs) by definition carry at least one driver mutations that increase cell growth advantage. It is challenging to identify signal of positive selection in CDGs that differentiate them for passenger genes harboring only random passenger mutations. Because of the high cost of experimental studies of gene functions, computational predictive algorithms become crucial to assess the evidence of candidate CDGs in a cohort of sequenced cancer samples.

**Fig. 1:**
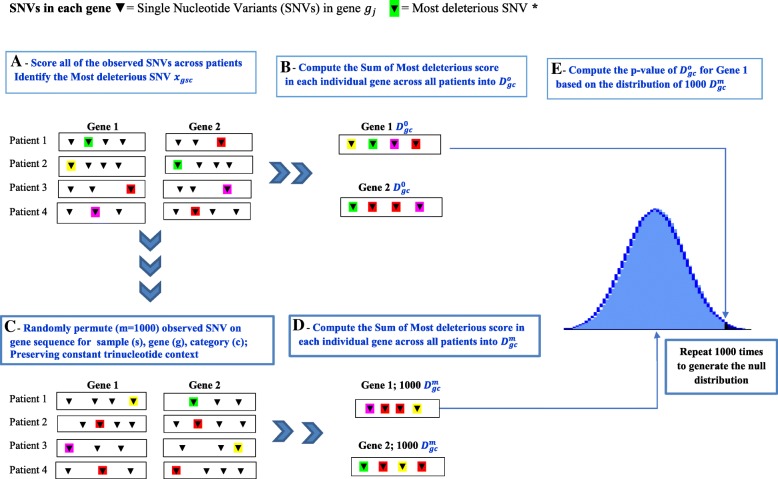
SMDS, a gene-based permutation method for the detection of candidate driver genes. The steps are shown from **a** to **e**

Here we introduce a gene-based permutation model (dubbed Sum of Most Deleterious Score or SMDS) to predict cancer CDGs in light of the pioneering InVeX method [4], a random permutation algorithm. Our algorithm infers enrichment of functional variants at each gene locus (Fig. 1) and applied it to predict the CDGs of breast and lung cancer. Unlike the InVEx approach which utilizes only one functional predictive method for missense SNVs (PolyPhen-2) [5], our algorithm leverages seven different scoring systems through a permutation based model. In addition, this new method covers both coding and non-coding regions of genes in order to infer new CDGs. We assume that for a cancer sample, one pathogenic driver mutation on a CDGs is enough to cause cancer, but different sample may have different pathogenic mutations in different driver genes. This implies that for each driver gene, only a small proportion of the samples may carry driver mutations. The power of our method to detect such driver gene depends on how different the deleteriousness prediction scores of the driver mutations compared to the artificial mutations we randomly imposed on to the gene. Given a list of observed SNVs from a cohort of cancer patients (samples), for each gene, the permutation approach randomly samples the position of each observed SNVs along a given gene sequence maintaining the trinucleotide context. Next, it identifies the Most Deleterious Score (MDS) per gene and sample (a monochromic measure of deleteriousness ranging from 0 to 1; and the larger the score, the more likely the variant is deleterious). Then, it tallies for each gene and across patients the SMDS 1000 times to build a null distribution and finally it computes an empirical *p*-value by comparing the observed SMDS against the null distribution of simulated SMDS.

## Results

### Comparison of the functional prediction scores

We have curated somatic mutations data from primary whole genome samples of two cancer types including 278,152 SNVs from 119 breast cancer patients and 468,348 SNVs from 24 lung cancer patients. These individual datasets belong to a large published dataset containing both whole-genome sequencing (WGS) and whole-exome sequencing data (WES) data [6]. After filtering out intergenic SNVs, the functional effect of these SNVs were scored using seven predictive methods: CADD [7], DANN [8], Fathmm-MKL coding and noncoding [9], MetaLR [10], SPIDEX [11] and VEST3 [12]. A summary of the seven predictive scores is presented in Additional file 1: Table S1.

First, we used the pairwise Pearson correlation coefficient (*r*) to measure the relationship between any pairs of the seven scores (Additional file 1: Table S2). Two pairs of scores (Fathmm-MKL coding and Fathmm-MKL noncoding, MetaLR and VEST3) were highly correlated (*r* ≥ 0.7); while six pairs of scores show medium correlation (0.4 < *r* < 0.7) and the remaining 13 pairs have a lower correlation (*r* ≤ 0.4) for breast and lung cancer. Additional file 1: Figure S1 presents UPGMA (Unweighted Pair Group Method with Arithmetic Mean) dendrograms clustering of scores according to their pairwise scores distance between scores measured by 1- *r*.

### Analysis of Most deleterious scores and sum of Most deleterious scores

We next identified the MDS(***X***_***gsc***_) of each score in each gene for each sample. We also computed the SMDS (${\boldsymbol{D}}_{\boldsymbol{gc}}^{\boldsymbol{o}}$) for each gene across all samples. Our analysis included 19,835 protein coding genes in breast cancer and 20,047 protein coding genes in lung cancer after the annotation process. For four pan-genome mutation predictive tools (CADD, DANN, Fathmm-MKL coding and Fathmm-MKL noncoding), each gene has a SMDS ${\boldsymbol{D}}_{\boldsymbol{gc}}^{\boldsymbol{o}}$> 0.0; meaning that at least one observed SNV in those genes was scored. However, for the three missense and splicing mutation scoring methods (MetaLR, SPIDEX and VEST3), because a higher proportion of SNVs are not missense or splicing mutations there are multiple genes with SMDS ${\boldsymbol{D}}_{\boldsymbol{gc}}^{\boldsymbol{o}}=$0.0. For instance, only 17 to 22% and 46 to 49% of the genes have ${\boldsymbol{D}}_{\boldsymbol{gc}}^{\boldsymbol{o}}$> 0.0 for missense scores (MetaLR and VEST3) and SPIDEX, respectively (Additional file 1: Figure S2).

### Finding candidate driver genes

We applied our gene-based permutation model to 19,835 and 20,047 protein coding genes identified from the annotation process (see Methods) for the breast and lung cancer data, respectively. We define candidate driver genes as those with *p*-values less than or equal to 0.01 (p-value ≤0.01). We found that a long list of genes met that criteria including 942 (4.7%) unique genes for breast and 796 (4.0%) for lung cancer (Table 1). Depending on the individual predictive method used in the permutation model: 0.8 to 1.3% genes showed statistically significant results for breast cancer and 0.6 to 0.9% genes for lung cancer. Additional file 1: Figure S3 presents the null distribution of TP53 (*p*-value < 0.01), a known breast cancer gene, and SLC1A2 (*p*-value = 0.195), a non-cancer gene, for the CADD score.

**Table 1:** Candidate driver genes positively selected (*p*-value ≤0.01) by each permutation model and their percentage (in brackets) of all genes tested for breast and lung cancer data

Permutation model	CADD	DANN	Fathmm-MKL coding	Fathmm-MKL noncoding	MetaLR	SPIDEX	VEST3	Unique genes	Total genes
Breast	263 (1.3)	158 (0.8)	178 (0.9)	184 (0.9)	174 (0.9)	178 (0.9)	171 (0.9)	942 (4.7)	19,835
Lung	121 (0.6)	142 (0.7)	138 (0.7)	149 (0.7)	178 (0.9)	171 (0.9)	164 (0.8)	796 (4.0)	20,047

### *P*-value distributions of the seven permutation models

**Fig. 2:**
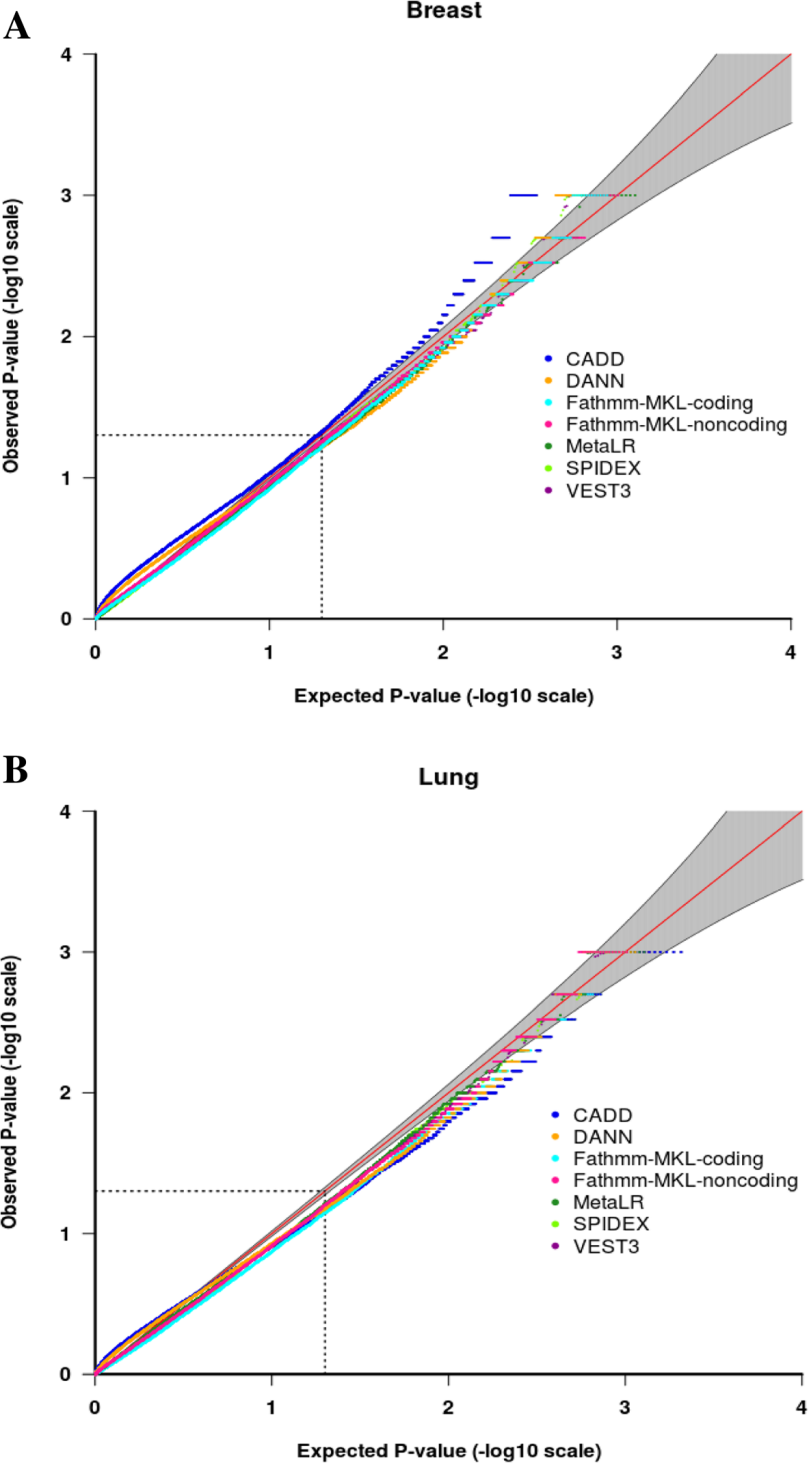
Quantile–quantile (QQ) plots of *p*-values comparing the observed distribution of p-values (y - axis) to the expected *p*-values of a null distribution (x - axis) for 19,835 breast cancer genes (Panel **a**) and 20,047 lung cancer genes (Panel **b**). The red line represents the expectation under the null. The grey area depicts the 95% confidence

The performance of the *p*-value distribution of the seven permutation models were evaluated using quantile–quantile (QQ) plots, which displays the relationship between the observed *p*-values to the expected uniform distribution of *p*-values under the null hypothesis. For breast cancer (Fig. 2a), most QQ plots for the individual permutation models show that the majority of genes fit the null expectations and only a small proportion of genes having a smaller p-value than expected. Indeed, the deviation of *p*-values from the expected distribution of *p*-values; as observed outside the 95% confidence interval (grey shading) at the top tail suggest candidate genes. Individual QQ plot for each independent model are shown in Additional file 1: Figure S4. The same trend was observed for the p-values for lung cancer genes (Fig. 2b and Additional file 1: Figure S5).

### Agreement in predicting candidate driver genes by the seven independent permutation models

**Fig. 3:**
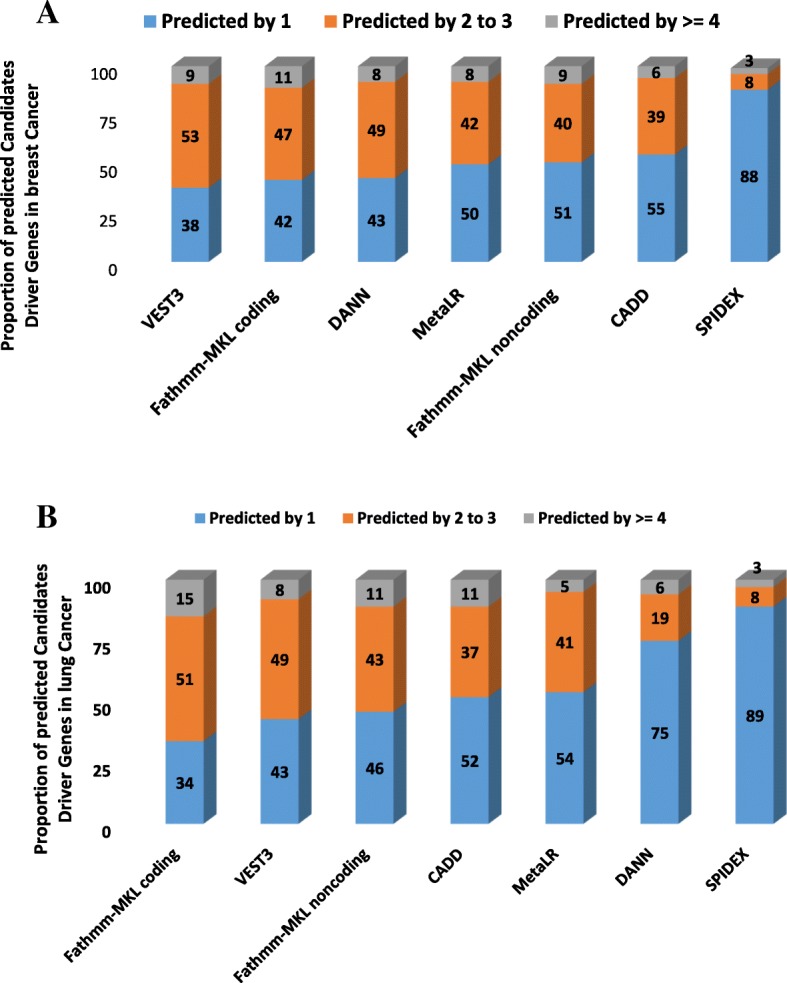
Proportion of breast cancer candidate driver genes (Panel **a**) and lung cancer genes (Panel **b**) predicted by one, two to three or more than three permutation models

To compare the predictions of the seven permutation models, we collected 942 unique genes positively selected (p-values ≤0.01) by each model from breast cancer and 796 genes for lung cancer patients. We started by assessing the agreement between all the seven models for breast cancer (Additional file 2: Table S3) and lung cancer (Additional file 2: Table S4). We identified the proportion of selected genes that were unique to each predictor or commonly chosen by two to three, or by more than three other permutation models for breast cancer (Fig. 3a) and lung cancer (Fig. 3b). For the pan-genome scores and across the two cancer tissues types, more agreement can be seen among CADD, DANN, Fathmm-MKL coding, and Fathmm-MKL noncoding compared to the three predictors scoring only coding or splice regions. For instance, in breast cancer, we found that taken individually each score selected roughly half of the genes (55, 43, 42 and 51%) unique to its own; whilst the remaining half were also selected by other scores. On the other hand, VEST3 has less unique genes (38%) compared to other predictors; MetaLR was comparable to the pan-genome scores (50%), whereas for SPIDEX most of its selected genes (88%) were unique to its own. In lung cancer (Fig. 3b) the same trend is observed among pan-genome scores that selected roughly one third to half of the genes unique to its own; whilst the remaining half to two third were also selected by other scores. VEST3 and MetaLR scores have unique genes comparable to the pan-genome scores; whereas for SPIDEX the vast majority of genes selected genes (89%) were unique to their own respectively breast and lung cancer.

**Fig. 4:**
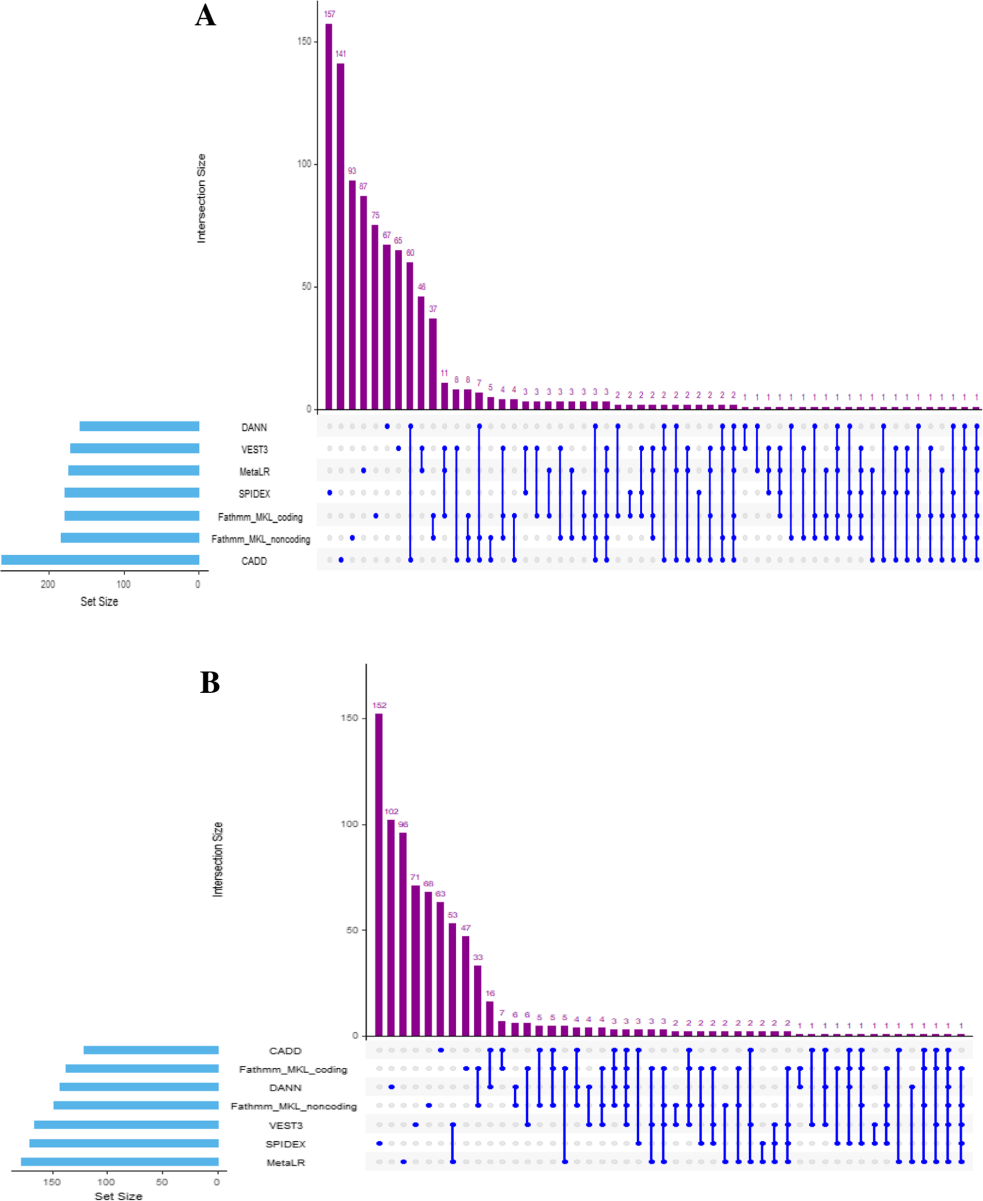
Comparison of the candidate driver genes predicted by seven models with 119 breast cancer samples (Panel **a**) and 24 lung cancer samples (Panel **b**). UpSet plot showing the intersection of overlap sets between the CDGs predicted by 7 permutation models (sets). Horizontal bars (Set Size) next to the tool names represent the total number of CDGs selected by each set. Vertical bars (Intersection Size) are annotated by connected blue dots reflecting the number of common CDGs detected by a specific combination of sets sorted by size

Figure 4 presents a matrix display of the intersection of the number of the breast candidate driver genes selected by the seven scores. Set intersections characterize common genes predicted by a set of scores. The blue circle in the matrix label scores that are part of the intersection. The results show that for breast cancer more genes were exclusively selected by only one score demonstrating the divergence of these methods: SPIDEX (157), CADD (141), Fathmm-MKL noncoding (93), MetaLR (87), Fathmm-MKL coding (75), DANN (67), and VEST3 (65). Moreover, fewer candidate genes were selected by a set of two scores CADD and DANN (60), MetaLR and VEST3 (46), Fathmm-MKL coding and Fathmm-MKL noncoding (37). The same trend is observed as the number of consensus scores increased till seven. We noted that intersecting the scores resulted in very few overlapping protein coding genes. There was one common gene (TP53) selected by all seven scores; 2 genes (GRIN1, XG) by six scores; 6 genes (TAF1L, MAP 3 K1, PIK3CA, OTOP1, PSMA4, FZD3) by five scores; and 13 genes by four scores (KMT2C, RTDR1, MICAL2, CBFB, SHBG, CDH10, C9orf135, GABRR1, ODAM, PHTF2, GANC, MAP 2 K4, FUNDC2) and 50 genes by three scores (Fig. 4a and Additional file 2: Table S3).

For lung cancer, there was one common gene (DLX4) selected by six scores; 4 genes (TP53, CCT7, ST6GAL2 and RBM10) by five scores; 16 genes (LY6G6E, STK11, MUSTN1, NF1, FBXW7, OR7C1, SLC27A1, SIRPD, CTIF, CEP250, LPA, RYR1, QRSL1, CHD3, KCNMB3) by four scores and 27 genes by three scores (Fig. 4b and Additional file 2: Table S4).

Next, we considered the agreement between the four models with pan-genome scores (CADD, DANN, Fathmm-MKL coding, and Fathmm-MKL noncoding). For breast cancer, these four scores were able to predict 583 unique genes (62%) out of the total 942 candidate genes. DANN detected fewer driver candidates (158) and CADD detected the highest number (263) (Table 1). Overall, 7 genes were commonly predicted by the four models (Additional file 1: Figures S6A and S7A). More genes were exclusively predicted by only one predictive model demonstrating the divergence of these models: CADD (156), Fathmm-MKL noncoding (100), Fathmm-MKL coding (97), and DANN (69). We found there was more agreement between some of these scores. We observed a higher number of genes selected by a combination of CADD and DANN (65), and Fathmm-MKL coding and Fathmm-MKL noncoding (48) and that was also noted in lung cancer. This higher correlation is expected as CADD and DANN shared the same training data, as well as Fathmm-MKL coding and noncoding.

We also assessed the agreement between the remaining three models for breast cancer. Two of these independent models only score missense mutations in coding regions (VEST3 and MetaLR) whereas SPIDEX cover exonic and intronic regions near splicing sites. Across the two cancer tissue types, they have provided the MDS (${\boldsymbol{D}}_{\boldsymbol{gc}}^{\boldsymbol{o}}$) for only 17 to 49% of the genome (Additional file 1: Figure S2). We found that for breast cancer, these three predictors selected 437 unique cancer candidate driver genes (46%) out of the 942 candidate driver genes. They have consensus on 5 genes (TP53, RAB37, ODAM, FZD3 and FUNDC2) (Additional file 1: Figures S6B and S7B). More genes were exclusively selected by only one predictor demonstrating the divergence of these methods: SPIDEX (167), MetaLR (99), and VEST3 (90) (Additional file 1: Figure S3B). We observed many shared predicted genes (70) between MetaLR and VEST3 (Additional file 1: Figure S7B).

For lung cancer, the four models with pan-genome scores (CADD, DANN, Fathmm-MKL coding, and Fathmm-MKL noncoding) selected 419 unique genes (53%) out of the total 796 candidate genes. As shown in Additional file 1: Figure S8A and S9A, they commonly predicted 7 genes (LY6G6E, TP53, STK11, MUSTN1, RBM10, DLX4 and CCT7). The remaining three models (MetaLR, VEST3 and SPIDEX) selected 435 unique cancer candidate driver genes (55%) out of the 796 candidate driver genes. Additional file 1: Figures S8B and S9B show that they have consensus on 5 genes (KCNC3, SPINK14, KCNMB3, CHD3, and ST6GAL2).

### Intersection of the predicted candidate driver genes for breast and lung cancer with the Cancer genes census

**Fig. 5:**
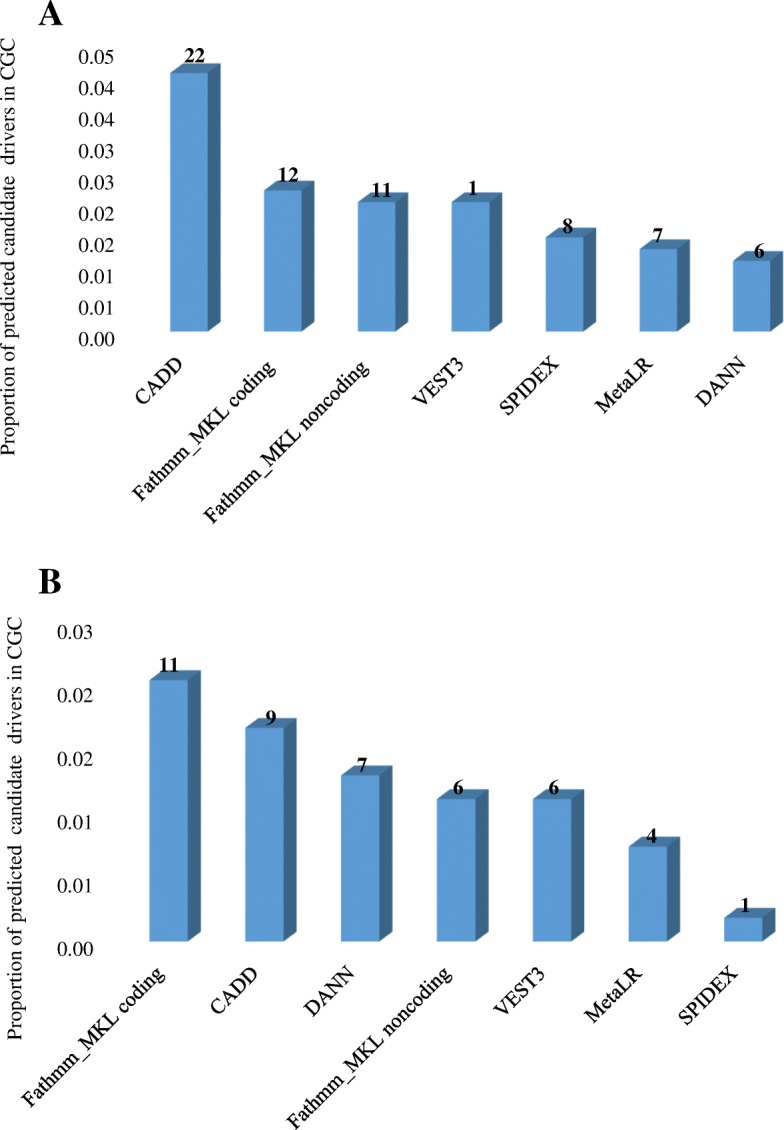
Proportion of 534 genes in the Cancer Gene Census predicted by each permutation model from breast cancer (Panel **a**) and lung cancer (Panel **b**). The number of predicted driver genes is on top of each bar

The CGC currently lists 534 genes (with somatic mutations) causally associated with different types of cancers. Among them 32 genes have been implicated in breast cancer. If considering only genes predicted by five or more scores, the seven predictive permutation models selected a total of 10 protein coding genes for breast cancer (Table 2). Among them, TP53 was selected by all seven scores and is known to cause breast cancer. Two other genes, PIK3CA and MAP 3 K1, which were selected by five scores, are also associated with breast cancer according to CGC. MAP 2 K4 were also selected by five scores and is a known to be associated with other cancer types. The remaining six genes (GRIN1, XG, TAF1L, OTOP1, PSMA4, and FZD3) are not listed in CGC. Additional file 1: Table S5 shows that all together these models were able to predict 15 (47%) of known breast cancer genes. Additional file 1: Table S6 lists the *p*-values of 32 breast cancer genes where 38, 25, 19, 19, 19, 16, and 13% of CGC genes were selected by CADD, VEST3, Fathmm-MKL coding, MetaLR, SPIDEX, Fathmm-MKL noncoding and DANN permutation models respectively. We also found that a total of 37 candidates predicted by the seven permutation models for breast cancer have already been linked to other types of cancer (Additional file 1: Table S5). Figure 5a shows the fraction of the 534 genes in CGC predicted by each model.

**Table 2:** Candidate driver genes predicted by five or more permutation models for breast and lung cancer

Number of shared predictive models	Number of genes predicted	Genes names overlapping with breast or lung cancer genes in CGC	Genes names overlapping with other cancer genes in CGC	Gene names not in CGC
Breast				
7	1	TP53		
6	2			GRIN1, XG
5	6	PIK3CA, MAP 3 K1	MAP 2 K4	TAF1L, OTOP1, PSMA4, FZD3
Lung				
7				
6	1			DLX4
5	4	TP53, RBM10		CCT7, ST6GAL2

In CGC, twelve genes with somatic mutations are causally associated with lung cancer. A total of 5 protein coding genes were predicted by five or more scores in this study. Among them, TP53 and RBM10 were selected by five scores are known to cause lung cancer according to CGC. The remaining three genes (DLX4, CCT7, and ST6GAL2) are not listed in CGC. Additional file 1: Table S7 shows that all together the seven models were able to predict 2 (6%) of known lung cancer genes. Additional file 1: Table S8 lists the *p*-values of 12 lung cancer genes found in CGC. Figure 5b shows the fraction of the 534 genes in CGC predicted by each model. We found that a total of 22 candidates predicted by the seven permutation models for lung cancer have already been linked to other types of cancer (Additional file 1: Table S7).

### Overall performance of the seven deleteriousness prediction scores

The overall performance of the seven permutation models was evaluated using the following three criteria including the number of genes predicted by each model, overlap with the CGC and the model consensus. These criteria were recently recommended by Tokheim et al. [13] for assessing the performance of driver gene prediction method in absence of a gold-standard method. The model consensus is the fraction of predicted candidate driver genes selected by two or more other predictors (Fig. 3a and b). The overlap with the CGC represent the fraction of the 534 genes in CGC predicted by each model (Fig. 5a and b). In this study, the top ranked scores for breast cancer are VEST3, Fathmm-MKL coding and CADD. For lung cancer, Fathmm-MKL coding, CADD, and VEST3 outperformed the other (Table 3).

**Table 3:** Performance comparison of the seven permutation models on breast and lung cancer

Method	Number of Significant genes	Overlap with CGC	Method consensus	CGC Rank	Consensus Rank	Average Rank
Breast cancer						
*CADD*	263	0.041	0.45	1	4	2.5
*DANN*	158	0.011	0.57	6	2	4
*Fathmm-MKL coding*	178	0.022	0.58	2	2	2
*Fathmm-MKL noncoding*	184	0.021	0.49	3	3	3
*MetaLR*	174	0.013	0.5	5	3	4
*SPIDEX*	178	0.015	0.12	4	5	4.5
*VEST3*	171	0.021	0.62	3	1	2
Lung cancer						
*CADD*	121	0.017	0.48	2	4	3
*DANN*	142	0.013	0.25	3	6	4.5
*Fathmm-MKL coding*	138	0.021	0.66	1	1	1
*Fathmm-MKL noncoding*	149	0.011	0.54	4	3	3.5
*MetaLR*	178	0.007	0.46	5	5	5
*SPIDEX*	171	0.002	0.11	6	7	6.5
*VEST3*	164	0.011	0.57	4	2	3

## Discussion

In this study we developed a function prediction based approach (SMDS) utilizing both coding and noncoding deleteriousness prediction scores for somatic SNVs observed in whole genome sequence data of cancer samples to identify potential cancer driver genes. We applied this approach to breast and lung cancer data sets using seven different functional prediction scores (CADD, DANN, Fathmm-MKL coding, Fathmm-MKL noncoding, MetaLR, SPIDEX and VEST3). A total of 942 unique gene were selected by the seven scores with *p*-values ≤0.01 in our permutation tests for breast cancer. Among them, ten protein coding genes were selected by five or more scores, which have higher likelihood to be true cancer driver genes. Among the ten genes, the well-known cancer driver gene TP53 is the only gene selected by all seven scores. PIK3CA, MAP 3 K1 and MAP 2 K4 are selected by five scores and known to be associated with breast cancer or other cancer types. Six new candidate genes were identified by at least five of the seven scores but not listed in the CGC: GRIN1, XG, TAF1L, OTOP1, PSMA4 and FZD3. However, there are some evidence supporting their involvement in cancer development.

For lung cancer, a total of 796 unique gene were selected by the seven scores with p-values ≤0.01 in our permutation tests. Among them, five protein coding genes were selected by five or more scores. Two are known lung cancer genes (RBM10 and TP53). The fewer number of know cancer genes predicted here for lung cancer may be attributed to the small sample size (24 samples compared to 119 samples in breast cancer) and higher level of passenger mutations (468,348 SNVs for lung cancer samples compared to 278,152 SNVs for breast cancer samples). Three new candidate genes identified by at least five of the seven scores are not listed in the CGC (DLX4, CCT7 and ST6GAL2). There are also evidence supporting their involvement in cancer development.

### PSMA4

The results of a recent study showed that mRNA high expression level of PSMA4 in multiple cancer types were significantly associated with worse prognostic in breast cancer, gastric cancer and HER2-negative gastric cancer; whereas they were correlated with better prognostic in lung adenocarcinoma [14]. A previous functional study reported the gene was involved in promoting cancer cell proliferation and apoptosis; and it was labeled as a “strong candidate mediator” associated with lung cancer susceptibility [15]. PSMA4 polymorphism has been associated to lung cancer risk in Chinese Han population [16]. The gene was also overexpressed in colorectal cancer patients and was significantly correlated with metastasis development and worse prognosis [17].

### TAF1L

This gene was previously reported as the fourth significantly mutated gene among 20 protein kinase genes rated by probability of harboring at least one mutation [18]. It was later identified as a potential driver gene with clinical relevance in melanoma cancer samples [19]. Interestingly, the authors found that it was recurrently mutated in pan-negative melanoma samples (without mutations in known melanoma cancer driver genes). The gene was found to be disrupted by frameshift mutations in Gastric and Colorectal Cancers [20] and because of its known involvement in apoptosis induction and cell cycle regulation; they hypothesized that the presence of frameshift mutations could decrease the cell death and therefore lead to higher survival of cancer cells in gastric and collateral cancer patients.

### GRIN1

Recent studies have demonstrated the role GRIN1 plays in tumorigenesis. One study [21] analyzed expression of GRIN1 in 12 different human tumor cell lines and concluded it was present in 9 of them including breast cancer. The gene is a calcium regulating tumor suppressor that was reported as one of the six hyper mutated genes impacting dysregulation of the glutamate signaling pathways in melanoma [22]. Functional mutations (loss of function) in this gene have been linked to tumor growth, proliferation and survival in melanoma. Another study [23] reported that functional receptors from this gene were crucial for maintaining tumor cell growth and viability in breast and could by target for the development of therapeutic drugs.

### FZD3

This gene was previously described as an oncogene and a probable therapeutic target gene [24]. The gene was found to be overexpressed in multiple cancer types including lung, leukemia, myeloma, lymphoma and sarcoma. A study assessing its clinical significance in colorectal cancer concluded that FZD3 was not only associated with carcinogenesis and progression; but also, its staining could be used as prognostic marker [25].

### OTOP1

This gene has been associated to diverse type of cancer including esophageal adenocarcinoma, pancreas, Melanoma, Lung, and prostate [26]. It was frequently mutated in lung cancer cell line genomes [27] and pancreatic tumors [28], but it was not conclusively classified as a driver gene.

### XG

This gene is known to be associated to lower survival and tumor invasiveness in Ewing’s Sarcoma (EWS) patients and was described as a biological marker for EWS [29, 30].

### DLX4

The methylation of DLX4 was strongly associated with high risk of recurrence and poor prognostic survival in lung cancer patients [31]. The gene can drive tumor progression in ovarian cancer through the NF-κB pathway by activating a regulatory factor cell surface molecule CD44 [32]. It was also found to promote ovarian cancer by inducing the expression of iNOS, an enzyme that stimulates angiogenesis [33].

### ST6GAL2

DNA methylation of ST6GAL2 has been proposed as a cancer biomarker for screening and detection. A recent study had linked ST6GAL2 hypermethylation to cervical intraepithelial neoplasia grade 3 or worse [34]. ST6GAL2 upregulation may be implicated to growth and proliferation in invasive ductal carcinoma (IDC) [35]. In follicular thyroid carcinoma, up-regulated ST6GAL2 in advanced cells and its co-expression with LncRNA HCP5 was strongly associated to cell proliferation, migration, invasiveness and angiogenesis [36].

### CCT7

This gene has been recently identified as a potential biomarker for endometrial carcinoma [37]; the gene was found to be highly expressed in a proteomic analysis comparing endometrial carcinoma and normal precarious tissues. According to the same authors, CCT7 has been linked to multiples cancer (neck cancer, adenocarcinoma, carcinoma squamous cell, neoplasms, malignant neoplasms and lymphoma) and health conditions (necrosis, staphylococcal scalded skin syndrome and Hodgkin disease). CCT7 was also identified as a biomarker linked to late stage colorectal cancer in a protein interaction sub-networks analysis for early tumorigenesis comparing normal and late stage colon cancer tissues [38]. A study comparing levels of mRNA expression during overall survival in glioblastoma multiform (GBM) human patients and protein expression during development of the macaque rhesus brain discovered eight signature genes including CCT7 that were higher expressed in early brain development, were associated with overall survival of in GBM patients and have the potential for drug target therapy [39].

## Conclusion

In this paper we discussed a gene-based permutation approach (SMDS) that functionally interrogates the whole genomes of cancer patients to identify potential candidate driver genes. We have performed a comprehensive analysis to predict CDGs by applying the SMDS method to breast and lung cancer data and comparing the scores of seven popular functional predictive methods. Each individual SMDS was able to identify a set of potential CDGs. We intersected CDGs predicted by at least five of the seven SMDS models and obtained a list of well-known cancer genes reported in the CGC and also novel CDGs that are worth further investigation. Our study also showed the advantages of utilizing pan-genome deleteriousness prediction scores in function prediction based methods for identifying cancer driver genes. Although for breast cancer the best performed score is tied between a missense prediction score, VEST3, and a pan-genome score, Fathmm-MKL coding, for lung cancer, on the other hand, two pan-genome scores, CADD and Fathmm-MKL coding performed better than missense prediction scores. Considering the pan-genomes scores’ performances are at least comparable to missense prediction scores yet provide complementary information, they shall be included in function prediction based approaches for detecting CDGs, as demonstrated in this study.

## Methods

### Cancer mutation data collection

We curated somatic SNVs primary from the whole genome sequence data of two cancer tissue types (278,152 SNVs from 119 breast cancer patients and 468,348 SNVs from 24 lung cancer patients) of a large published dataset containing both whole-genome sequencing and whole-exome sequencing data [6]. These somatic mutation data were obtained as follows as described by the authors. Normal DNA samples and tumor samples of the same individual were sequenced. All somatic mutations data of each sample was then combined to generate its mutational catalog.

### Individual predictive methods

The seven individual scores included in our analysis were initially developed to prioritize functional mutations and all are non-cancer specific (Additional file 1: Table S1). CADD [7], is a meta-annotation tool that contrasts existing genomic variation to simulated genomic variation. It uses a linear kernel SVM to differentiate benign variants from deleterious variants (binary classification) by integrating the information of diverse functional annotations (evolutionary conservation, regulatory and transcript information, and protein-level scores) into a single score. It scores the deleteriousness of SNVs as well as insertion/deletions variants for both coding and non-coding regions. DANN [8] algorithm uses the same features set and training data as CADD to train a DNN which scores every possible SNVs in order to capture non-linear relationships among features. Fathmm-MKL [9] predicts the functional, molecular and phenotypic consequences of SNVs of both coding and noncoding regions. It uses a MKL classifier to combine ten different features groups including functional annotations from ENCODE and nucleotide-based conservation measures. Two scores were produced by this method based different training features: coding and noncoding. Both scores are pan-genome. MetaLR [10] scores the deleteriousness of missense SNVs. It combines individual scores from ten predictors including nine scores (SIFT [40], PolyPhen-2 [5], GERP++ [41], MutationTaster [42], Mutation Assessor [43], Fathmm [44], LRT [45], SiPhy [46], PhyloP [47]) and the maximum minimum frequency observed in the 1000 genomes populations into one ensemble score using a LR model. SPIDEX [11] algorithm is a Bayesian ensemble of DNN trained with RNA sequencing data. It scores all synonymous, missense and nonsense exonic SNVs, as well as intronic SNVs that are up to 300 nt from splice junctions. VEST3 [12] method integrated 86 features from SNVBox [48] (conservation scores, amino acid residue substitution scores, UniProtKB annotations, and predicted local protein structure) to predict the functional significance of missense mutations.

### Annotation and scoring of variants

SNV annotation was done through the WGSA pipeline [49]. Three different annotation software (ANNOVAR [50], SnpEff [51] and VEP [52]) with two different databases (RefSeq [53] and Ensembl [54]) were used to functionally annotate all the SNVs. After the annotation process, we retrieve the following four VEP gene annotation fields: VEP_ensembl_Consequence, VEP_ensembl_Gene_Name, VEP_ensembl_Protein_ID, and VEP_ensembl_CANONICAL. We filter only protein coding genes from other genes using the VEP_ensembl_Protein_ID. Because some genes have more than one transcript, we restricted our analysis to the canonical transcripts. The region of each gene was defined as from 5 kb upstream to 5 kb downstream. All seven scores (CADD, DANN, Fathmm-MKL noncoding and Fathmm-MKL coding, VEST3 and MetaLR) for each SNV were annotated through WGSA [49]. To make the individual scores comparable to one another CADD and SPIDEX scores were rescaled to rank between 0 and 1. Because not all the seven scoring systems are applicable to the entire genome; three methods including MetaLR and VEST3 (score only missense SNVs) and SPIDEX (score only part of exon and intron regions) had a high number of variants with missing values. The lowest score (0.0) was used for those variants.

### A gene-based permutation model

We designed a gene-based permutation model to prioritize cancer candidate driver genes in light of the InVeX method [4]. We applied this new approach to compare the performance of seven predictive methods (CADD, DANN, Fathmm-MKL noncoding and Fathmm-MKL coding, MetaLR, SPIDEX and VEST3) that measure the functional effect of SNVs in both coding and non-coding regions of the genome. For a cancer tissue type and assuming there are total of (*G*) genes*,* (*S*) samples, and(*C*) categories (predictive scores of each individual predictive method); first, we identify the MDS (*X*_*gsc*_) of each observed SNV, for each sample (*s*), gene (*g*) and category/score (*c*). MDS is a monochromic measure of deleteriousness ranging from 0 to 1; and the larger the score, the more likely the variant is deleterious). Second, we compute the SMDS where all the MDS per sample (*s*) are tallied for each gene (*g*) and each category (c) into ${D}_{gc}^o={\sum}_{s=1}^S{X}_{gsc}$. Third, we generate within-gene null distribution to compute *p*-values in the observed data. Basically, for each sample (*s*), gene (*g*) and category (*c*), the gene-based permutation approach randomly permuted the position of the each observed SNV along the gene sequence, preserving the trinucleotide context in each sample (i.e. for a ACG > ATG SNV we randomly choose another ACG trinucleotide sites from the gene sequence, from 5 kb stream to 5 kb downstream, and “move” the ACG > ATG SNV there) and this is done 1000 times. Next, the newly “simulated” SNVs were annotated and scored and each set is known as a trial (total number of trials =1000). Fourth, for each trial set, we identify the MDS (${X}_{gcs}^m$) and compute the SMDS (${D}_{gc}^m={\sum}_{s=1}^S{X}_{gsc}$) for the simulated data. Fifth, we compute individual p-value (${P}_{gc}^o$) for each gene (*g*) and for each category (*c*) for the observed ${D}_{gc}^o$ based on the empirical null distribution of the simulated 1000 ${D}_{gc}^m$ scores (m = 1000). ${P}_{gc}^o$was defined as the percentage of the simulated ${D}_{gc}^m$ scores equal or greater than the observed ${D}_{gc}^o.$

### Plotting

The QQ-plot of p-values showing the distribution of p-values were produced in R using the function pQQ from the Haplin library [55]. The intersection between the candidate driver genes predicted by each individual predictive model were found using UpSetR [56]. The Veen diagrams were drawn in the website http://bioinformatics.psb.ugent.be/webtools/Venn/. The dendrogram (UPGMA cluster analysis) were produced in R using the package UPGMA, (https://www.rdocumentation.org/packages/phangorn/versions/2.4.0/topics/upgma).

## Additional files

Additional file 1:**Figure S1.** UPGMA dendrogram comparing the seven prediction scores for breast cancer (Panel A) and lung cancer (Panel B). **Figure S2.** Percentage of genes with Sum of Most Deleterious Scores ${D}_{gc}^o$ > 0.0 covered by each of the 7 predictive models in for breast and lung cancer data. **Figure S3.** Null distribution of the permuted Sum of Most Deleterious Scores ${D}_{gc}^m$ for the CADD score in TP53 (*p*-value = 0.000) well-known breast cancer gene; and SLC1A2 (p-value = 0.195) gene not associated with cancer. The red dots and lines indicate the observed values 6.7 for TP53 and 3.7 for SALL4. **Figure S4.** Quantile–quantile plot of the observed *p*-values for breast cancer genes (y - axis) against the expected *P* values of a null distribution (x - axis). The red line represents the expectation under the null hypothesis. The grey area depicts the 95% confidence interval. **Figure S5.** Quantile–quantile plot of the observed p-values for lung cancer genes (y - axis) against the expected *P* values of a null distribution (x - axis). The red line represents the expectation under the null hypothesis. The grey area depicts the 95% confidence interval. **Figure S6.** Proportion of breast candidate driver genes predicted by one, two to three, and more than three permutation models: **Panel A**- Agreement between CADD, DANN, Fathmm-MKL coding and Fathmm-MKL noncoding; **Panel B**- Agreement between MetaLR, SPIDEX and VEST3. **Figure S7.** Comparison of breast candidate genes driver predicted by seven independent permutation models. **Panel A**- Venn diagram of candidate driver genes predicted by CADD, DANN, Fathmm-MKL coding, and Fathmm-MKL noncoding. **Panel B**- Venn diagram of candidate driver genes predicted MetaLR, SPIDEX and VEST3. **Figure S8.** Proportion of lung candidate driver genes predicted by one, two to three, and more than three permutation models: **Panel A**- Agreement between CADD, DANN, Fathmm-MKL coding and Fathmm-MKL noncoding; **Panel B**- Agreement between MetaLR, SPIDEX and VEST3. **Figure S9.** Comparison of lung candidate driver genes predicted by seven independent permutation models. **Panel A**- Venn diagram of candidate driver genes predicted by CADD, DANN, Fathmm-MKL coding, and Fathmm-MKL noncoding. **Panel B**- Venn diagram of candidate driver genes predicted MetaLR, SPIDEX and VEST3. **Table S1.** Summary of methods for scoring somatic mutations (SNVs) deleteriousness. **Table S2**. Pearson’s correlation Coefficients between the seven predictive scores for breast cancer (Upper Triangle) and lung cancer (Lower Triangle). **Table S5.** Breast cancer candidate driver genes predicted by one or more permutation models. **Table S6.** *P*-values for each permutation model for the 32 breast cancer genes in Cancer Genes Census. **Table S7.** Lung cancer candidate driver genes predicted by one or more permutation models. **Table S8.** *P*-values for each permutation model for the 12 lung cancer genes in Cancer Genes Census. (PDF 1721 kb)

Additional file 2: **Table S3.** Predicted breast cancer driver genes by the seven permutation models. **Table S4.** Predicted lung cancer driver genes by the seven permutation models. (XLSX 43 kb)

## Abbreviations

CADD: Combined Annotation Dependent Depletion
CDGs: Cancer Driver Genes
CGC: Cancer Gene Census
DNN: Deep Neural Network
Fathmm-MKL: Functional Analysis through Hidden Markov Models - Multiple Kernel Learning
InVeX: Introns Vs Exons
LR: Logistic Regression
MDS: Most Deleterious Score
MKL: Multiple Kernel Learning
SMDS: Sum of Most Deleterious Score
SNVs: Single Nucleotide Variants
SVM: Support Vector Machine
UPGMA: Unweighted Pair Group Method with Arithmetic Mean
VEP: Ensembl Variant Effect Predictor
VEST3: Variant Effect Scoring Tool
WES: Whole-Exome Sequencing
WGS: Whole-Genome Sequencing
WGSA: Whole Genome Sequencing Annotator
