# Supplementary material for: Comparison of different functional prediction scores using a gene-based permutation model for identifying cancer driver genes
Source: BMC Med Genomics. 2019 Jan 31;12(Suppl 1):22. doi: 10.1186/s12920-018-0452-9 (PMC6357357; doi:10.1186/s12920-018-0452-9)
Supplement: Supplementary file 1 — Figure S1. UPGMA dendrogram comparing the seven prediction scores for breast cancer (Panel A) and lung cancer (Panel B). Figure S2. Percentage of genes with Sum of Most Deleterious Scores ${D}_{gc}^o$Dgco > 0.0 covered by each of the 7 predictive models in for breast and lung cancer data. Figure S3. Null distribution of the permuted Sum of Most Deleterious Scores ${D}_{gc}^m$Dgcm for the CADD score in TP53 (p-value = 0.000) well-known breast cancer gene; and SLC1A2 (p-value = 0.195) gene not associated with cancer. The red dots and lines indicate the observed values 6.7 for TP53 and 3.7 for SALL4. Figure S4. Quantile–quantile plot of the observed p-values for breast cancer genes (y - axis) against the expected P values of a null distribution (x - axis). The red line represents the expectation under the null hypothesis. The grey area depicts the 95% confidence interval. Figure S5. Quantile–quantile plot of the observed p-values for lung cancer genes (y - axis) against the expected P values of a null distribution (x - axis). The red line represents the expectation under the null hypothesis. The grey area depicts the 95% confidence interval. Figure S6. Proportion of breast candidate driver genes predicted by one, two to three, and more than three permutation models: Panel A- Agreement between CADD, DANN, Fathmm-MKL coding and Fathmm-MKL noncoding; Panel B- Agreement between MetaLR, SPIDEX and VEST3. Figure S7. Comparison of breast candidate genes driver predicted by seven independent pe [file 12920_2018_452_MOESM1_ESM.pdf]

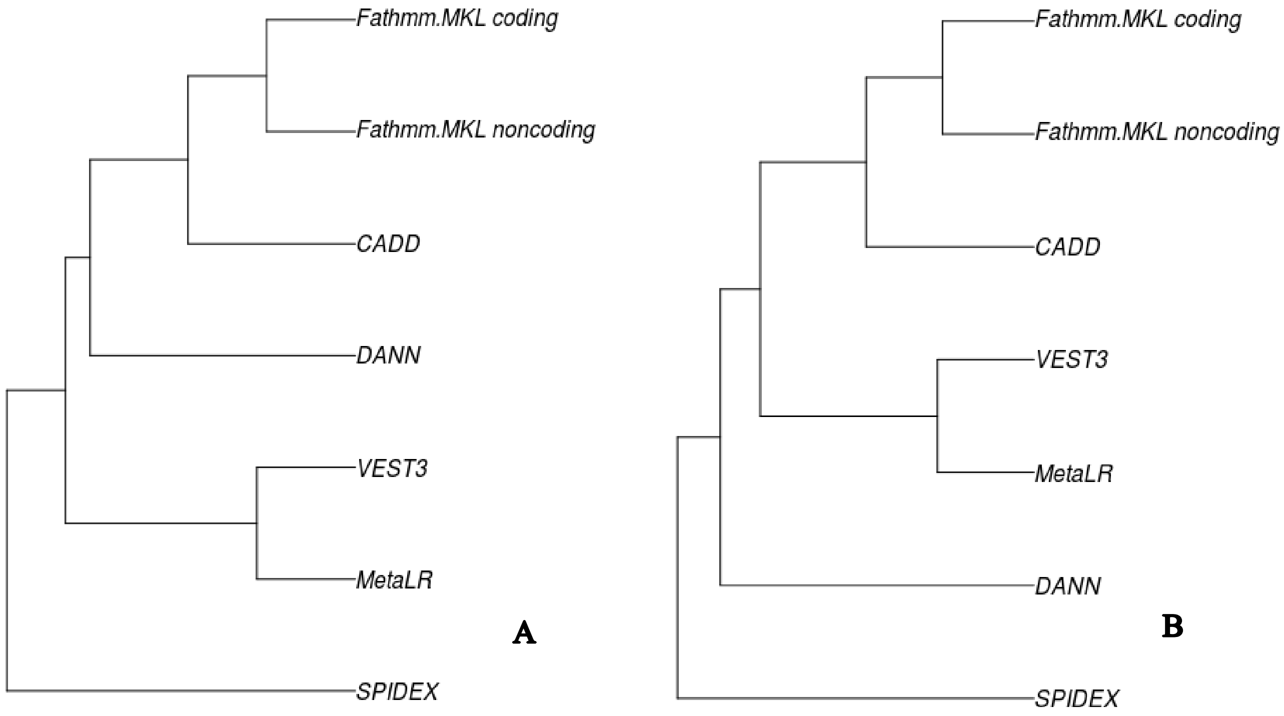

Fig. S1 *UPGMA dendrogram comparing the seven prediction scores for breast cancer (Panel A) and lung cancer (Panel B).*

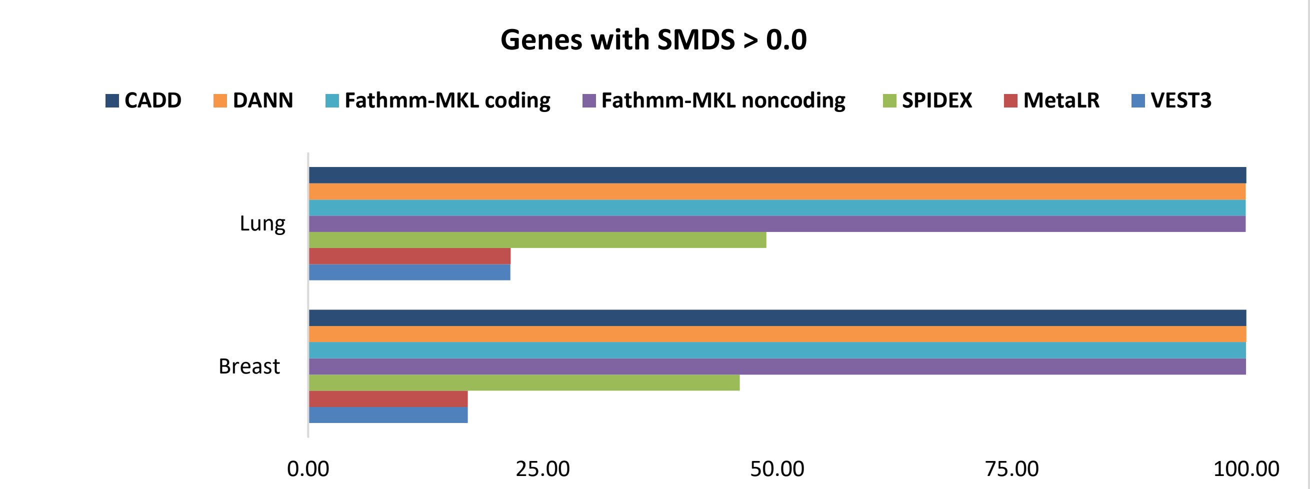

	Breast	Lung
CADD	100.00	100.00
DANN	100.00	99.94
Fathmm-MKL coding	99.99	99.93
Fathmm-MKL noncoding	99.99	99.93
SPIDEX	46.00	48.84
MetaLR	17.00	21.60
VEST3	17.00	21.55

Fig. S2 *Percentage of genes with Sum of Most Deleterious Scores* $\boldsymbol{D^{o}_{gc}}$ *> 0.0 covered by each of the 7 predictive models in for breast and lung cancer data.*

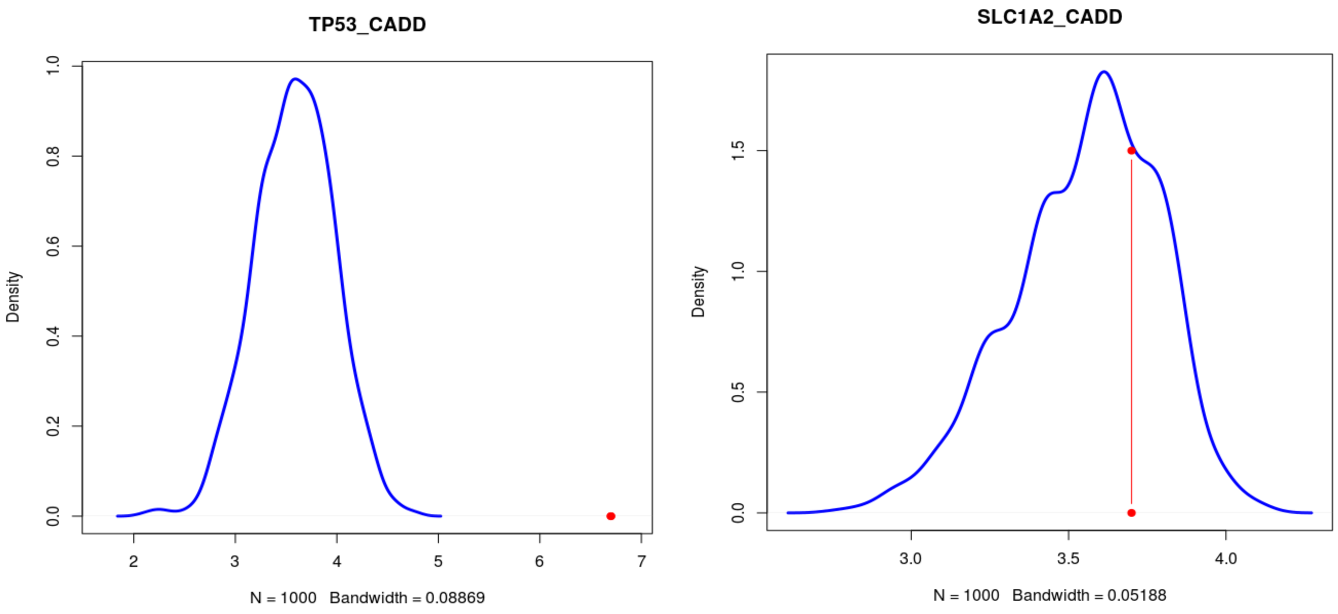

Fig. S3 *Null distribution of the permuted Sum of Most Deleterious Scores* $\boldsymbol{D^{m}_{gc}}$ *for the CADD score in TP53 (p-value = 0.000) well-known breast cancer gene; and SLC1A2 (p-value = 0.195) gene not associated with cancer. The red dots and lines indicate the observed values 6.7 for TP53 and 3.7 for SALL4.*

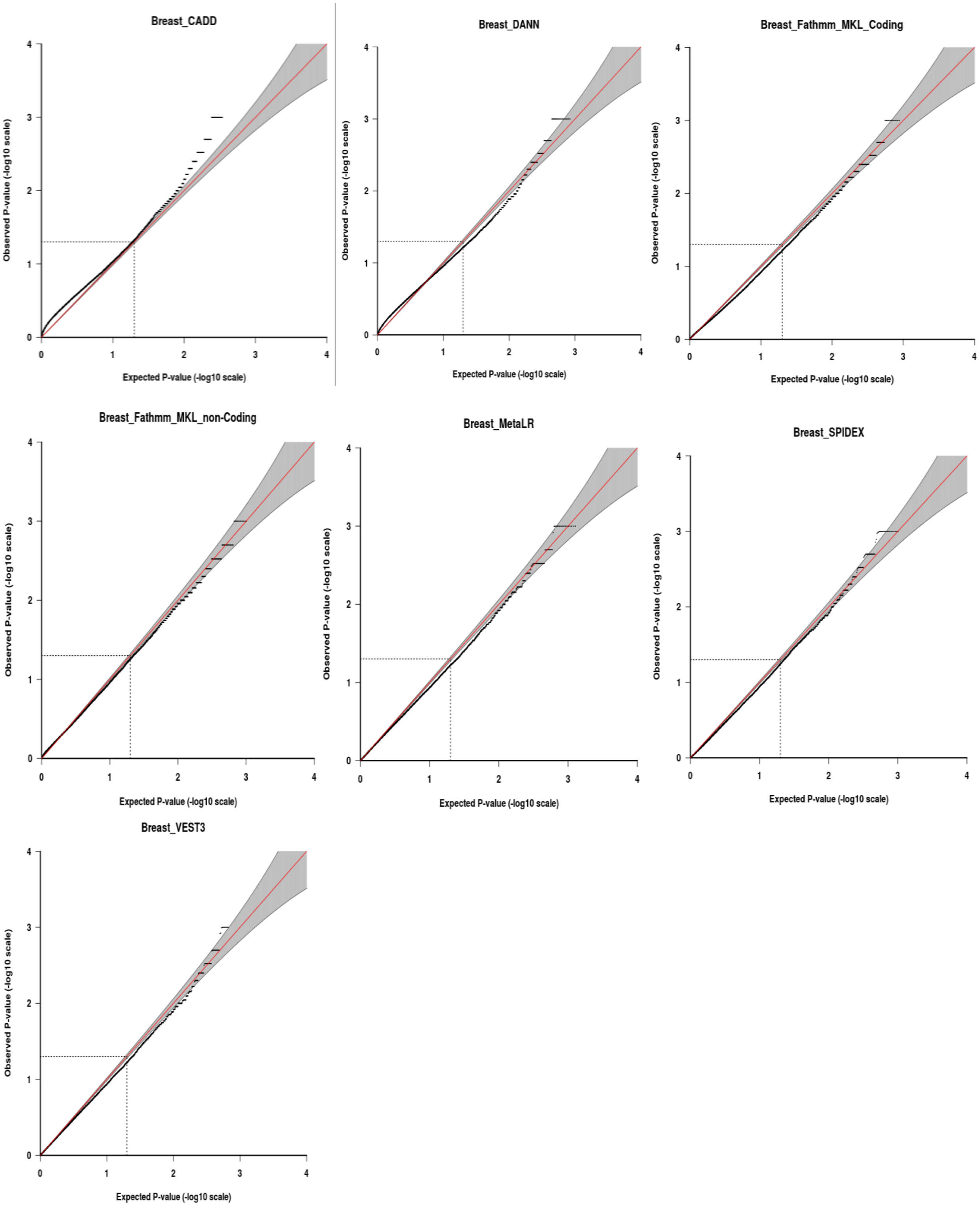

Fig. S4 *Quantile–quantile plot of the observed p-values for breast cancer genes (y - axis) against the expected P values of a null distribution (x - axis). The red line represents the expectation under the null hypothesis. The grey area depicts the 95% confidence interval.*

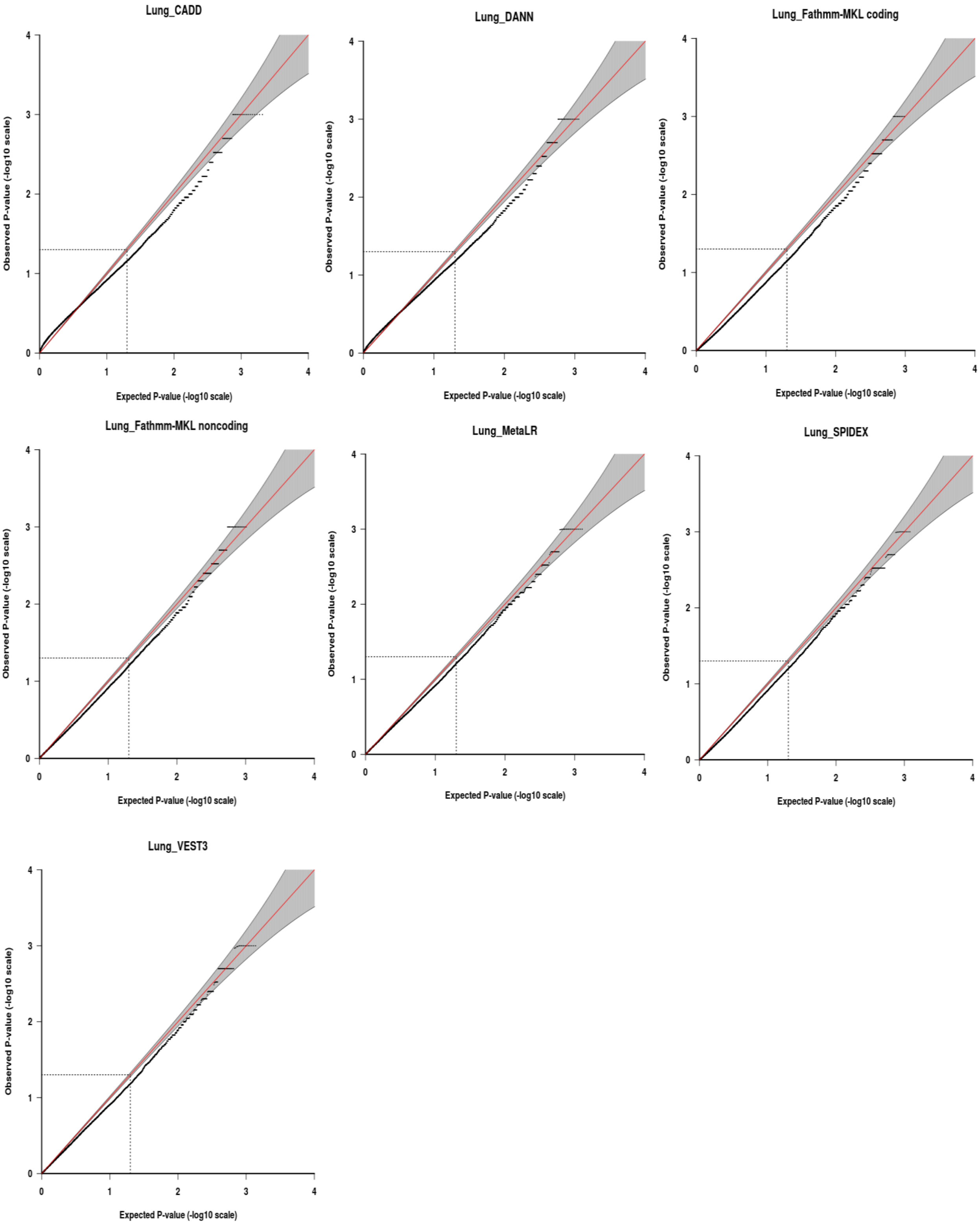

Fig. S5 *Quantile–quantile plot of the observed p-values for lung cancer genes (y - axis) against the expected P values of a null distribution (x - axis). The red line represents the expectation under the null hypothesis. The grey area depicts the 95% confidence interval*

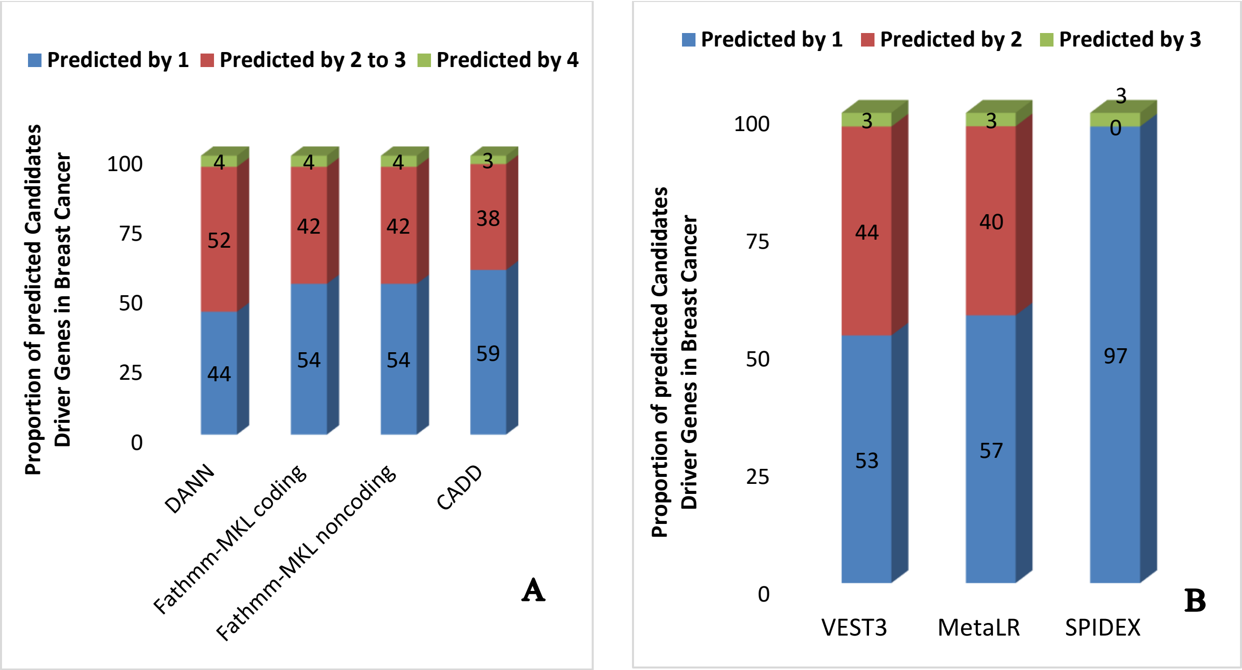

Fig. S6 *Proportion of breast candidate driver genes predicted by one, two to three, and more than three permutation models:* ***Panel A****- Agreement between CADD, DANN, Fathmm-MKL coding and Fathmm-MKL noncoding;* ***Panel B****- Agreement between MetaLR, SPIDEX and VEST3.*

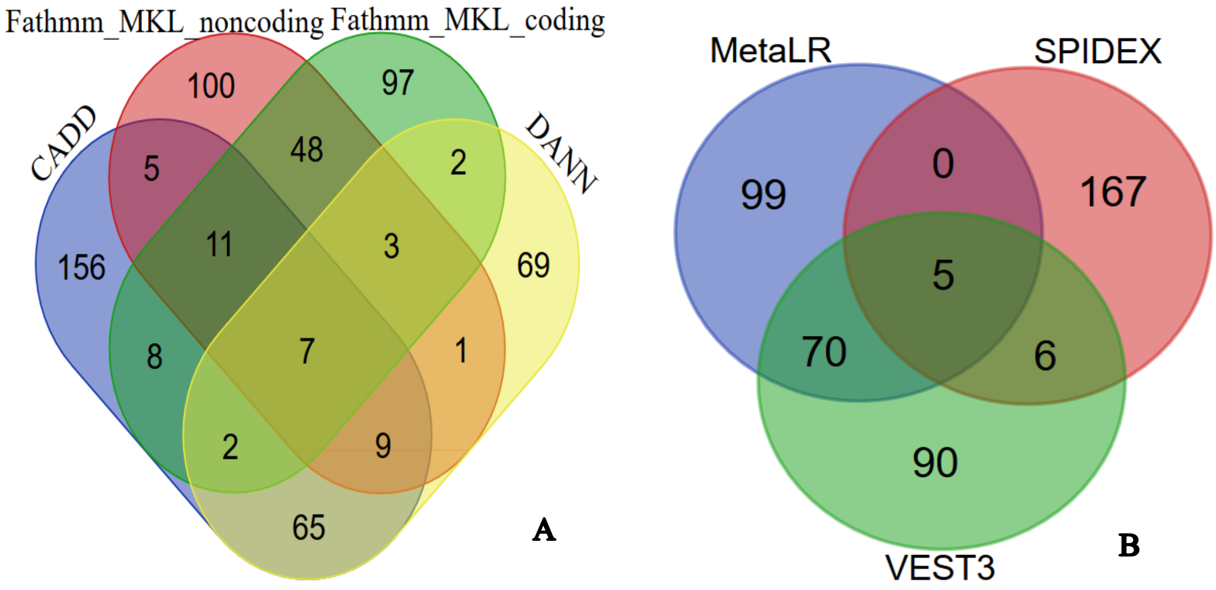

Fig. S7: *Comparison of breast candidate genes driver predicted by seven independent permutation models.* ***Panel A****- Venn diagram of candidate driver genes predicted by CADD, DANN, Fathmm-MKL coding, and Fathmm-MKL noncoding.* ***Panel B****- Venn diagram of candidate driver genes predicted MetaLR, SPIDEX and VEST3.*

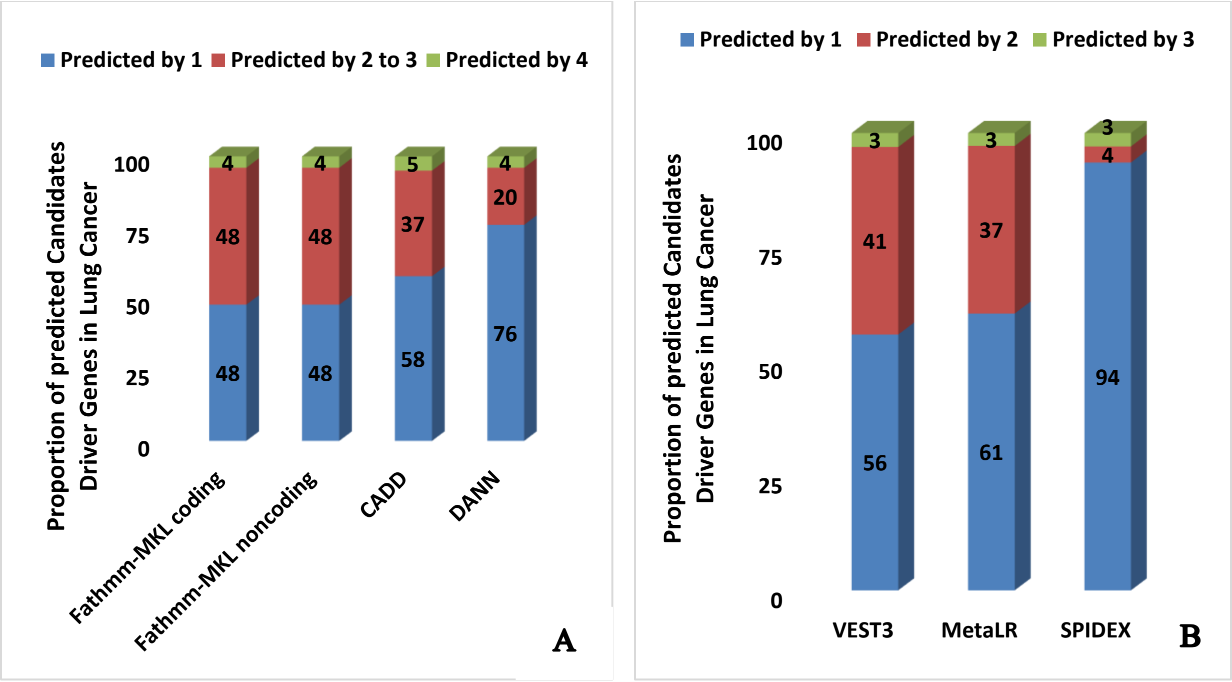

Fig. S8: *Proportion of lung candidate driver genes predicted by one, two to three, and more than three permutation models:* ***Panel A****- Agreement between CADD, DANN, Fathmm-MKL coding and Fathmm-MKL noncoding;* ***Panel B****- Agreement between MetaLR, SPIDEX and VEST3.*

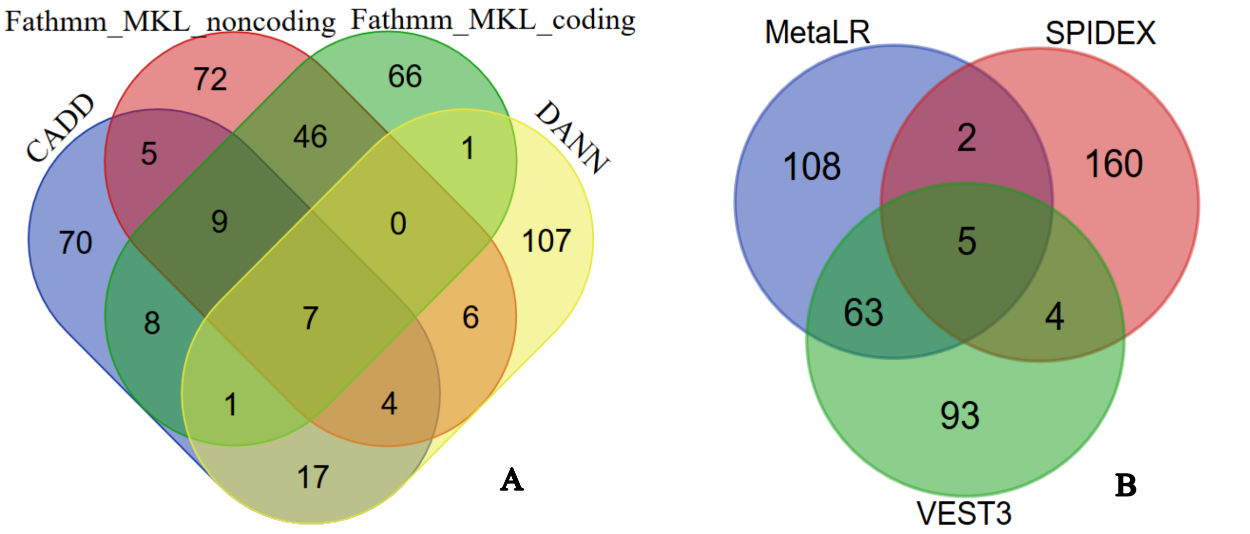

Fig. S9: *Comparison of lung candidate driver genes predicted by seven independent permutation models.* ***Panel A****- Venn diagram of candidate driver genes predicted by CADD, DANN, Fathmm-MKL coding, and Fathmm-MKL noncoding.* ***Panel B****- Venn diagram of candidate driver genes predicted MetaLR, SPIDEX and VEST3.*

Table S1: Summary of methods for scoring somatic mutations (SNVs) deleteriousness

Method name	Category	Score used for analysis	Description and statistical method used
**CADD**	Ensemble score	(Score-Min) / (Max −Min)	Uses a **linear kernel support vector machine** (SVM) to combine 63 distinct annotations [949 features such as functional annotations, conservation, and gene-model information (retrieved from VEP, ENCODE project and UCSC genome)] into a single metric (C-score). Larger CADD Phred-scaled scores are more likely to have damaging effect. Scores variants in both coding and non-coding regions relative to all possible substitutions of the human genome.<br>C-score ≥ 10 means top 10% as probable functional, C-score ≥ 20 means top 1% variants and finally C-score ≥30 means most deleterious top 0.1% human variants
**DANN**	Ensemble score	Score	Uses a **"deep neural network" (DNN)** and training data from CADD to score every possible SNV in both coding and noncoding regions. Variations that are predicted deleterious are annotated at 3 different score levels.
**Fathmm-MKL**	Deleteriousness prediction	Score	Uses a **kernel learning multiple (MKL**) classifier to combine 10 different features groups including functional annotations from ENCODE and nucleotide-based conservation measures. Scores are p-values ranging from 0 to 1. Scores > 0.5 are predicted to be deleterious.
**MetaLR**	Ensemble score	Score	Uses a **logistic regression algorithm** for prediction of deleterious missense SNVs. It combines individual scores from ten predictors (SIFT, PolyPhen-2 HDIV, PolyPhen-2 HVAR, GERP++, MutationTaster, Mutation Assessor, FATHMM, LRT, SiPhy, PhyloP) and the maximum frequency observed in the 1000 genomes populations. The scores ranges from 0 to 1 and a larger score means the SNV is more likely to be damaging.
**SPIDEX**	Ensemble score	(Score-Min) /(Max −Min)	Uses a **Bayesian ensemble of deep neural networks** to predict the functional effect of SNVs near splicing sites. It estimates the percentage of transcripts with the exon spliced in (PSI or Ψ) based on the analysis of 1393 sequence features from each exon and RNA-sequencing data from 16 human tissues. Splicing patterns are based on the probability of PSI scores ranging from low (≤33%), medium (34–66%) to high (≥67%).
**VEST3**	Deleteriousness prediction	Score	Uses **random forest classifier** to integrate 86 features from SNVBox (conservation scores, amino acid residue substitution scores, UniProtKB annotations, and predicted local protein structure) and predict the functional significance of missense mutations. Score ranges from 0 to 1. Larger scores are more likely to cause functional change.<br>Can also compute a gene-based prioritization score.

Tools for annotation of SNVs are displayed. These tools will compute different scores and each SNV (mutation) will end up with seven annotations.

Table S2: Pearson's correlation Coefficients between the seven predictive scores for breast cancer (Upper Triangle) and lung cancer (Lower Triangle)

	SPIDEX	***CADD***	***DANN***	***Fathmm-MKL noncoding***	***Fathmm-MKL coding***	***MetaLR***	***VEST3***
SPIDEX	-	0.20	0.08	0.19	0.24	0.24	0.28
CADD	0.20	-	0.52	0.62	0.61	0.42	0.49
DANN	0.08	0.51	-	0.37	0.30	0.17	0.20
Fathmm-MKL noncoding	0.18	0.63	0.36	-	**0.80**	0.28	0.33
Fathmm-MKL coding	0.23	0.62	0.28	**0.80**	-	0.37	0.45
MetaLR	0.24	0.41	0.15	0.28	0.37	-	**0.78**
VEST3	0.28	0.49	0.18	0.33	0.44	**0.78**	-

Table S5: Breast cancer candidate driver genes predicted by one or more permutation models.

Number of shared predictive models	Number of genes predicted	Number of genes and genes names overlapping with only breast cancer genes in CGC	Percentage overlapping with breast cancer genes in CGC	Number of genes and genes names overlapping with all cancer genes in CGC	Percentage overlapping with all cancer genes in CGC	Genes names overlapping with all cancer genes in CGC
7	1	1<br>TP53	100	1<br>TP53	100	
6	2					GRIN1, XG
5	6	2<br>PIK3CA, MAP3K1	33	3<br><br>MAP2K4, MAP3K1, PIK3CA	50	TAF1L, OTOP1, PSMA4, FZD3
4	13	1<br>MAP2K4	8	3<br><br>KMT2C, CTCF, PTEN	23	RTDR1, MICAL2, SHBG, CDH10, GABRR1, C9orf135, ODAM, PHTF2, GANC, FUNDC2
3	50	1<br>CTCF	2	2<br><br>CBFB, RB1	4	
2	185	3<br>GATA3, RB1, CASP8,	2	6<br><br>NF1, EBF1, PPFIBP1, GATA3, KLF4, BCL6	3	
1	685	7<br><br>BRCA1, ERBB2, NCOR1, CDH1, TBX3, AKT1, KEAP1,	1	22<br><br>BRCA1, PREX2, DNMT3A, ERBB2, NCOR1, MLLT4, HOXC13, CREB3L2, CDH1, CUX1, SMARCE1, LIFR, KLF4, FAS, PRDM1, LMO1, EIF4A2, ERG, USP6, NR4A3, CDK12, KEAP1	3	
**Total**		**15**		**37**		

Table S6: P-values for each permutation model for the 32 breast cancer genes in Cancer Genes Census.

Genes	*CADD*	*DANN*	*Fathmm-MKL coding*	*Fathmm-MKL noncoding*	*MetaLR*	*SPIDEX*	*VEST3*
TP53	**0.000**	**0.000**	**0.000**	**0.000**	**0.000**	**0.000**	**0.000**
PIK3CA	**0.004**	**0.015**	**0.000**	**0.006**	**0.000**	0.053	**0.000**
GATA3	**0.005**	**0.019**	0.223	0.027	**0.010**	0.101	**0.004**
MAP2K4	0.037	0.102	**0.000**	**0.003**	**0.000**	**0.010**	**0.000**
CTCF	**0.001**	0.036	**0.001**	0.179	0.061	0.036	**0.005**
MAP3K1	**0.001**	**0.015**	**0.000**	**0.001**	**0.007**	0.222	0.222
RB1	**0.002**	0.040	0.020	0.141	**0.010**	**0.010**	**0.016**
AKT1	0.158	0.647	0.129	0.075	0.020	**0.008**	0.032
BRCA1	**0.007**	0.041	0.152	0.360	0.065	0.067	**0.010**
ARID1B	0.073	0.466	0.020	0.040	0.126	0.125	0.091
KEAP1	0.099	0.132	0.035	0.045	0.043	0.136	**0.009**
TBX3	**0.010**	0.146	0.107	0.115	0.160	**0.001**	0.153
ERBB2	**0.002**	0.047	0.071	0.076	0.047	0.070	0.022
CASP8	**0.010**	0.132	**0.006**	**0.005**	0.074	0.032	0.040
CDH1	**0.005**	0.153	0.059	0.096	0.841	0.471	0.635
FOXA1	0.050	0.081	0.147	0.100	0.074	0.222	0.116
BAP1	0.197	0.213	0.342	0.434	0.209	0.069	0.114
NCOR1	**0.002**	0.310	0.162	0.117	0.318	0.506	0.495
NOTCH1	0.035	0.170	0.344	0.120	0.466	0.823	0.626
ARID1A	0.259	0.599	0.468	0.655	0.525	0.861	0.958
CCND1	0.961	0.987	0.637	0.747	0.538	0.795	0.473
BRCA2	0.106	0.262	0.633	0.747	0.358	0.805	0.679
CDKN1B	0.649	0.679	0.841	0.839	0.899	0.594	0.691
EP300	0.092	0.123	0.229	0.186	0.098	0.235	0.092
ESR1	0.568	0.605	0.397	0.711	0.577	0.715	0.306
ETV6	0.732	0.979	0.833	0.726	0.400	0.196	0.733
MAP3K13	0.066	0.164	0.357	0.443	0.058	0.409	0.122
NTRK3	0.225	0.127	0.536	0.694	0.133	0.313	0.123
PBRM1	0.124	0.252	0.638	0.681	0.727	0.482	0.648
PPM1D	0.655	0.904	0.996	0.978	0.539	0.306	0.389
SALL4	0.833	0.903	0.778	0.613	0.877	0.763	0.727
SMARCD1	0.290	0.517	0.070	0.103	0.079	0.582	0.094
Percentage (%)	**38**	**13**	**19**	**16**	**19**	**15**	**25**

Table S7: Lung cancer candidate driver genes predicted by one or more permutation models.

Number of shared predictive models	Number of genes predicted	Number of genes and genes names overlapping with only lung cancer genes in CGC	Percentage overlapping with lung cancer genes in CGC	Number of genes and genes names overlapping with all cancer genes in CGC	Percentage overlapping with all cancer genes in CGC	Gene names not in CGC
**7**						
6	1					DLX4
5	4	2<br>TP53, RBM10	50	2<br>TP53, RBM10	50	CCT7, ST6GAL2
4	16			4<br>TP53, STK11, NF1, FBXW7	25	LY6G6E, STK11, MUSTN1, NF1, FBXW7, OR7C1, SLC27A1, SIRPD, CTIF, CEP250, LPA, RYR1, QRSL1, CHD3, KCNMB3
3	27			1<br>MAX	0.03	
2	152			4<br>PBRM1, MSN, CTNNB1, KEAP1	0.02	
1	599			12<br>CBL, BRD3, ERBB4, P2RY8, CSF3R, FCGR2B, PTPN11, ATP2B3, HMGA2, FAS, FGFR3, MTOR	0.02	
**Total**		**2**		**22**		

Table S8: P-values for each permutation model for the 12 lung cancer genes in Cancer Genes Census.

Genes	CADD	DANN	Fathmm-MKL coding	Fathmm-MKL noncoding	MetaLR	SPIDEX	VEST3
TP53	**0.001**	**0.003**	**0.000**	**0.000**	0.022	0.055	**0.010**
RBM10	**0.007**	**0.010**	**0.004**	**0.005**	0.078	0.164	**0.010**
RAD21	0.773	0.575	0.731	0.851	0.934	0.472	0.703
PTPN13	0.759	0.539	0.488	0.478	0.637	0.913	0.639
KRAS	0.318	0.200	0.349	0.196	0.621	0.922	0.588
NOTCH1	0.425	0.097	0.652	0.472	0.779	0.688	0.727
PTPRT	0.671	0.860	0.185	0.055	0.901	0.649	0.960
GRIN2A	0.293	0.594	0.688	0.308	0.623	0.526	0.610
MYCL	0.578	0.546	0.918	0.976	0.606	0.527	0.680
HIF1A	0.341	0.266	0.848	0.894	0.936	0.843	0.522
STRN	0.020	0.596	0.125	0.263	0.134	0.500	0.022
RB1	0.069	0.096	0.733	0.360	0.981	0.359	0.347
